# Gold Standard GFR measurement and GFR estimation in pediatric oncology – indications and limitations

**DOI:** 10.1007/s00467-025-07099-0

**Published:** 2025-12-28

**Authors:** Emil den Bakker, Martine F. Raphael, Arend Bokenkamp

**Affiliations:** 1 Department of Pediatric Nephrology, Amsterdam University Medical Center, Emma Children’s Hospital, Meibergdreef 9, NL-1105 AZ Amsterdam, The Netherlands; 2 Department of Pediatric Oncology, Emma Children’s Hospital, Amsterdam, The Netherlands

**Keywords:** Measured GFR, Estimated GFR, Creatinine, Cystatin C, Carboplatin, Malignancy, Children

## Abstract

Knowledge of glomerular filtration rate (GFR) is essential for the treatment and monitoring of children with a malignancy. Ideally, GFR is measured using the gold standard technique of renal inulin clearance. Still, this procedure is too invasive and time-consuming for routine clinical practice. Also, the armamentarium of markers suitable for GFR measurement (mGFR) has changed in the last decades, with iohexol clearance now being used in many countries, both in Europe and the US. For daily monitoring of kidney function, cystatin C has proven to be an important adjunct to serum creatinine, the standard marker of GFR. This review gives a comprehensive update on the indications and limitations of mGFR methods as well as the estimation of GFR from serum markers (eGFR) with special emphasis on children treated for a malignancy.

**Graphical abstract:**

**Figure:**
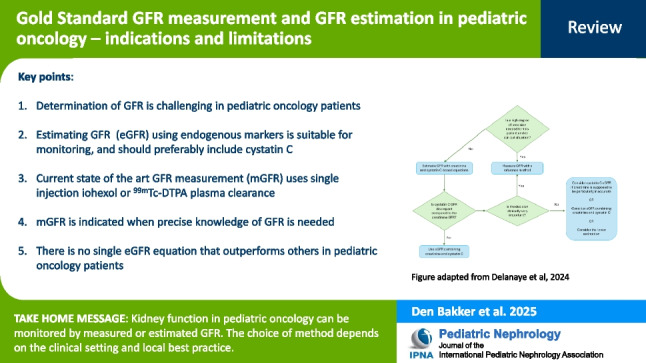
A higher resolution version of the Graphical abstract is available as Supplementary information

**Supplementary Information:**

The online version contains supplementary material available at 10.1007/s00467-025-07099-0.

## Background

Besides microalbuminuria, the glomerular filtration rate (GFR) is the key parameter when describing kidney health [1]. GFR quantifies kidney function and, as such, is important for the detection and follow-up of kidney injury and an important surrogate endpoint in many interventional studies.

Monitoring of kidney function is particularly important in children undergoing or in follow-up after treatment of a malignancy because they are at risk of kidney injury from cytostatic (e.g., cisplatin, ifosfamide, high-dose methotrexate) and adjuvant medication (e.g., aminoglycosides, anti-fungal medication, cidofovir), as well as radiotherapy and nephrectomy. Acute kidney injury is observed in 7 to 20% of adult cancer patients [2] and in 10 to 15% of children [3, 4] and may also be due to tumor lysis or dehydration from diarrhea and vomiting. Kidney injury may also manifest more insidiously – referred to as acute kidney disease [2, 5] – and progress to chronic kidney disease [6–10]. Knowledge of kidney function is also essential for drug dosing [11].

This review will give an update on the measurement of kidney function with special attention to children undergoing or in follow-up after treatment of a malignancy.

## The concept and methodology of measured GFR

GFR is the rate of fluid passage across the glomerular membrane. The concept of GFR and its measurement dates back to the seminal work by Homer Smith in the 1940s [12]. The GFR is measured by intravenous (iv) administration of an exogenous marker, which is excreted exclusively via glomerular filtration. Therefore, the marker must not be protein-bound, nor undergo tubular reabsorption or secretion, and not be eliminated by extra-renal metabolism. Inulin clearance is the gold standard technique for GFR measurement (mGFR) [12].

One must be aware that the *true* GFR is changing constantly and that measured GFR (mGFR) is an approximation of reality. GFR has a circadian rhythm and peaks in the daytime. GFR is also affected by renal blood flow and the glomerular capillary hydrostatic pressure. This is illustrated by the acute change in GFR observed after a protein or iv amino acid load: GFR can rise by up to 100% [13]. Therefore, GFR measurement protocols must be standardized with respect to timing, sufficient hydration, discontinuation of medication affecting GFR (e.g., ACE inhibitors or angiotensin receptor blockers, NSAID), and limited protein intake for at least 12 h prior to and during the test [14]. Variability in true GFR hampers comparison between different mGFR methods unless the measurements are performed simultaneously. This is illustrated by Rowe et al., who found an intraindividual variability of 6.7% in 4 consecutive weekly measurements of iohexol clearance in 20 adults [15], which is in line with a previous study using ^51^Cr-EDTA [16].

### Markers used for GFR measurement

Inutest 25%®, the polyfructosan preparation used for inulin clearance measurement, is not marketed any more. In the last decades, several other substances have been identified, which meet the characteristics of an exogenous GFR marker, i.e., iohexol [14], ethylenediaminetetraacetic acid (EDTA) [17], diethylenetriaminepentaacetic acid (DPTA) [18] and iothalamate [19]. While iohexol and iothalamate are iodine-containing contrast media, ^51^Cr-EDTA and ^99m^Tc-DTPA are radioactively labeled. For iothalamate, a radioactive preparation (^125^I-Iothalamate) is available, too. Characteristics of the different markers are summarized in Table 1.

**Table 1:** Characteristics of exogenous GFR markers

Marker	MW (Da)	Protein binding (%)	Extrarenal Clearance (ml/min/1.73 m^2^)	Radiation dose (MBq, adult)	Radiation t_1/2_	Measurement
Polyfructosan	3200	0	0	n.a	n.a	enzymatic
Iohexol (non-ionic)	821	1.5	0–6	n.a	n.a	HPLC, LC–MS/MS
Iothalamate (ionic)	614	9.6	4–10	n.a	n.a	HPLC, LC–MS/MS capillary electrophoresis
^51^Cr-EDTA	292	6–12	2–4	3	27 days	gamma camera
^99m^Tc-DTPA	393	5–11	10–13	10	6 h	gamma camera
^125^ J-Iothalamate (ionic)	614	9.6	4–10	10	60 days	gamma camera

Data from [30, 89–91]

MW; molecular weight, n.a.; not applicable, HPLC; high pressure liquid chromatography, LC–MS/MS; tandem mass spectroscopy.

While iohexol, iothalamate, and ^51^Cr-EDTA are stable and can be shipped for external quantification, measurement of ^99m^Tc-DTPA has to be performed immediately because of the rapid decay of ^99m^Tc. The pre-analytics and analytics of iohexol have been reviewed extensively in a recent consensus paper [14]. Iohexol is very stable and can also be measured in capillary blood samples [20]. In Sweden, an external quality assurance program has been set up for iohexol, which reported high reproducibility both in-house and between laboratories [21].

The armamentarium of GFR markers has shrunken since ^51^Cr-EDTA is no longer available for diagnostic purposes in Europe [22], while this marker never had FDA approval [23]. This has prompted a number of recent publications comparing simultaneous ^51^Cr-EDTA and ^99m^Tc-DTPA clearance studies [22, 24–27]. The paper by Gracia et al. [28] uses an elegant population pharmacokinetic approach to compare ^99m^Tc-DTPA and ^51^Cr-EDTA clearance by reversing the technique used for the development of eGFR equations, which will be discussed below.

### Measurement of GFR

The classic clearance technique with timed urine collection (referred to as *urinary clearance*) requires two iv-lines, an iv-loading dose, and subsequent continuous infusion. After an equilibration time of one hour, urine is collected for 30 min at least three times, and blood is drawn halfway through each collection period [29]. The GFR is calculated from marker excretion in timed urine collections divided by marker concentrations in serum. GFR is commonly normalized to the arbitrary body surface area of an adult (i.e. 1.73 m^2^).

*Urinary clearance* is a cumbersome procedure, and timed urine collections pose a special problem in children or patients with voiding disorders. This has prompted the development of methods to measure GFR without the need for urine collection. Of these, *plasma clearance* is the most widespread. This method calculates GFR from the disappearance kinetics after injection of the marker [30]. Unlike the other methods, it is not suitable for patients with edema or ascites, where sequestration of the tracer in inaccessible spaces results in an overestimation of the GFR [14]. ^*99mc*^*DTPA-renography* uses time-activity curves in the cortical region of the kidneys after tracer injection to determine GFR and also allows the function of both kidneys to be compared [18, 31]. Still, this method has proven imprecise in validation studies [30]. The *constant infusion technique* is based on the principle that GFR can be calculated as the ratio between the administrated tracer dose per unit of time (mg/min) divided by the plasma concentration in steady state. This has been described for iohexol [32], inulin [33], ^51^Cr-EDTA [22], ^99mc^DTPA [22] and iothalamate [34]. It requires very exact administration of the tracer for at least 4 to 5 h and can also be performed by subcutaneous administration on an outpatient basis [34].

### Calculation of plasma clearance

**Fig. 1:**
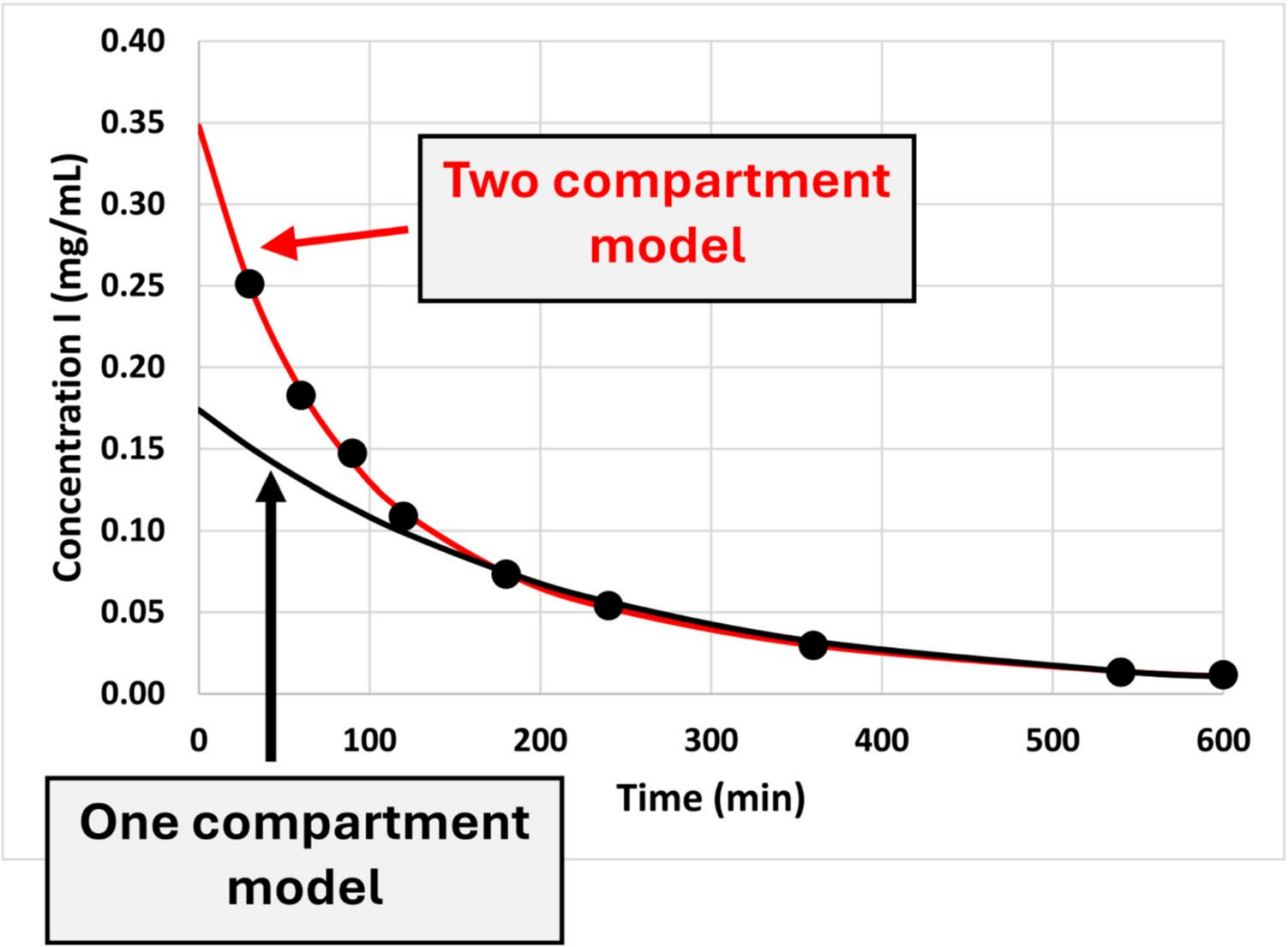
**Time course of marker concentration after a single injection.** In the two-compartment model (red) early sampling also captures the distribution phase while the one-compartment model (black) only uses late samples. The latter underestimates the real area-under-the concentration time curve (AUC), resulting in an overestimation of GFR. Therefore, a correction factor is required. From Pottel et al. [38], used with permission

While the calculation of GFR using the *urinary clearance* and the *constant infusion technique* is straightforward, the *plasma clearance* requires pharmacokinetic modeling. Following injection, the marker plasma concentration declines as a result of distribution in the extracellular compartment and glomerular filtration, and can be described by a two-compartment model (Fig. 1). Equilibration in the extracellular compartment dominates the pharmacokinetics in the first 1 to 2 h after injection and, in combination with glomerular filtration, leads to a rapid fall in marker concentrations (early phase). After equilibration, the rate of decline slows down as it reflects solely glomerular filtration (late phase, referred to as “slow GFR” in some publications).

The GFR is calculated as the dose injected divided by the area under the decay curve [30]. Optimally, 3–4 early and 3–4 late samples are required to calculate the full two-compartment model, yet for practicability reasons, this can be reduced to 2 early and 2 late samples as in the CKiD study [35]. To minimize patient discomfort, this can be further simplified by using a one-compartment model in which only late samples are used. As shown in Fig. 1, this model underestimates the AUC in the early phase, leading to an overestimation of GFR. Correction formulae for the GFR calculated from the one-compartment model were published by Bröchner-Mortensen [36] and more recently by Ng et al. [37]. While both formulae perform comparably at low and normal GFR, the Ng correction might be more suitable to detect hyperfiltration [38].

The choice of sampling timepoints is crucial for the accuracy of plasma clearance studies and depends on the expected GFR [39]. In a recent consensus statement on iohexol plasma clearance, sampling at 2 and at 4 h post injection is recommended for GFR above 60 ml/min/1.73 m^2^, at 3 to 7 h for GFR between 30 and 60, and at 4 and 8 h for GFR below 30 [14]. Although less invasive, the single sample technique [26] has a higher risk of error as it does not allow for any plausibility check and is not recommended in children [38].

### Comparing different mGFR methods

It has become common practice to refer to all methods measuring GFR using an external marker as “gold standard GFR”. Still, the true benchmark is the classical inulin clearance – which is no longer available. In the absence of this benchmark, comparison of different mGFR methods can only describe differences in bias and variability but cannot answer the question “Which method is best?”.

**Fig. 2:**
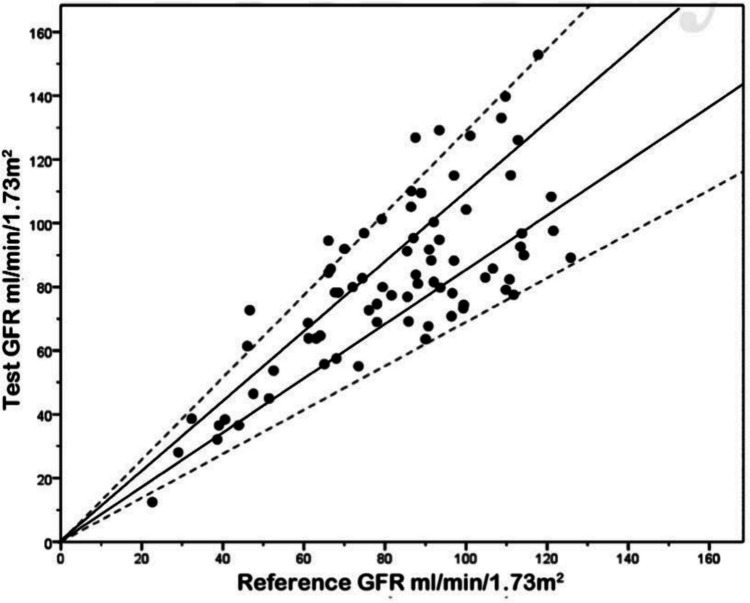
Accuracy P_10_ and P_30_ of GFR estimates. Distribution of paired results of the reference vs. the method under study. The dotted lines indicate measurements within ± 30% of the reference standard (i.e. P_30_ accuracy), measurements between the full lines are within ± 10% of the reference standard (i.e. P_10_ accuracy). The predictive uncertainty increases with higher GFR

Soveri et al. published a meta-analysis comparing the different mGFR techniques to classical inulin clearance [40]. They used stringent inclusion criteria (maximum interval between the mGFR measurements 48 h, at least 15 participants, and external validation of the algorithm used to calculate *plasma clearance*). Accuracy criteria were median bias below 5%, P_30_ accuracy at least 80%, and P_10_ accuracy of at least 50%. The concept of P_30_ and P_10_ accuracy is illustrated in Fig. 2. Based on a total of 1,367 paired measurements, the authors found strong or moderately strong evidence that the *urinary clearance* of iothalamate, the *urinary and plasma clearance* of ^51^Cr-EDTA, and the *plasma clearance* of iohexol were sufficiently accurate to replace classical inulin clearance. Limited evidence suggested that inulin *plasma clearance*, ^99m^Tc-DTPA *urinary clearance*, and iohexol *urinary clearance* had sufficient accuracy, too. Of note, all but inulin plasma clearance had P_30_ exceeding 90%. Although this is the largest and most thorough analysis comparing different mGFR techniques, the conclusion that ^99m^Tc-DTPA *plasma clearance* had insufficient accuracy was criticized because it was based on only 89 clearance measurements [41]. Since then, several studies have compared simultaneous ^99m^Tc-DTPA and ^51^Cr-EDTA *plasma clearance* and found good agreement. Vidal-Petiot reported a mean bias around 2 ml/min corresponding to about 3% of GFR, a P_30_ accuracy of 100%, and P_10_ accuracy of 92%, which was comparable to urinary clearance performed at the same time [27]. Using a single sample technique, Simonsen et al. observed some underestimation of ^51^Cr-EDTA of about 3 ml/min at low GFR, while the opposite was seen at high GFR [26]. These differences fall within the variability of *true* GFR and are considered non-significant for clinical decision making so that ^99m^Tc-DTPA *plasma clearance* is an acceptable alternative to ^51^Cr-EDTA clearance [24–28].

**Fig. 3:**
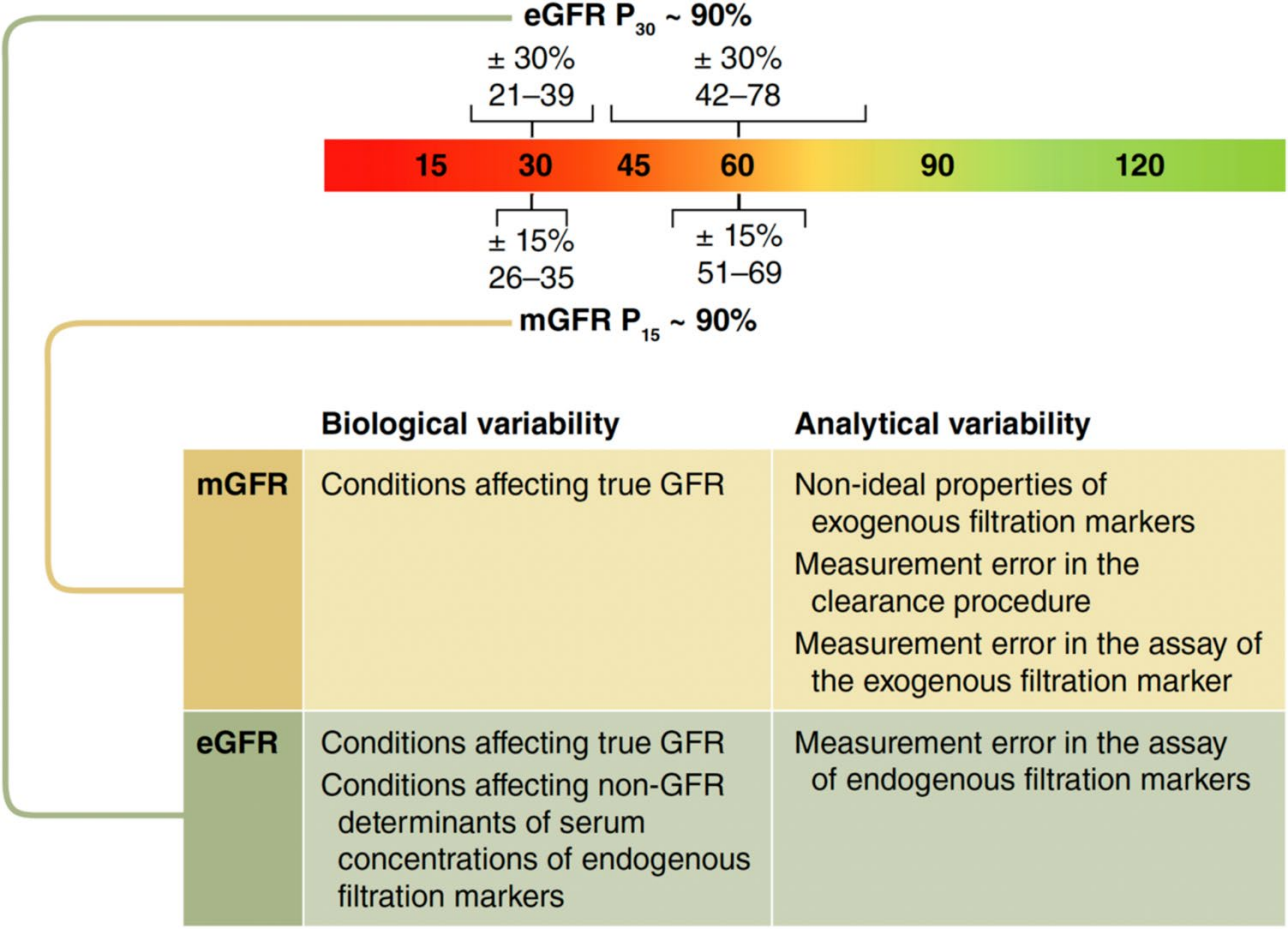
**Inaccuracy of mGFR and eGFR.** Sources and magnitude of error around measured glomerular filtration rate (mGFR) and estimated glomerular filtration rate (eGFR). From Inker et al. [64], used with permission

Therefore, anno 2025, iohexol and ^99m^Tc-DTPA *plasma clearance* can be regarded as the standard for GFR measurement when GFR estimation using endogenous GFR markers is regarded as unreliable [42] (Fig. 3). Adamson et al. stressed the need for standardization of mGFR protocols, in particular for drug dosing in oncological patients [43]. Such a consensus statement was recently published for plasma iohexol clearance in adults [14]. For children, Pottel and Schwartz advocate an iohexol *plasma clearance* protocol with the one-compartment model taking samples at 120, 180, 240, and 300 min after injection. For correction of the absence of the early samples, the Ng formula is recommended, followed by indexation to 1.73 m^2^ BSA as the final step [38].

Some clinicians still regard creatinine clearance calculated from 24-h urine collection as a substitute for mGFR. Endogenous creatinine clearance is insufficiently accurate and systematically overestimates GFR due to unpredictable tubular creatinine secretion [40]. In the past, inhibition of tubular creatinine secretion by administration of cimetidine was proposed [44, 45], but this technique is not sufficiently reliable and has not been widely adopted. Instead, estimated GFR (eGFR) based on serum concentrations of endogenous markers is recommended in international guidelines [46].

## Estimation of GFR

Rather than going through the cumbersome process of measuring GFR with an exogenous marker, in clinical practice GFR is usually estimated (eGFR) using serum levels of an endogenous marker. An endogenous marker should meet the requirements of an exogenous GFR marker with the additional prerequisite that it is produced at a constant rate [47]. Assuming a constant production rate, variation in serum level reflects changes in GFR. The most commonly used endogenous markers are creatinine and cystatin C. It should be kept in mind that equilibration in the volume of distribution of creatinine (total body water) and cystatin C (extracellular space) requires time and eGFR measurements lag behind the true kidney function [48].

Creatinine is a metabolite of creatine originating from muscle cells. Therefore, creatinine serum levels are influenced by muscle mass, the intake of boiled meat, or oral creatine supplements. As muscle mass increases during linear growth, creatinine production increases until young adulthood, most markedly in boys after puberty. This can be accounted for by relating individual creatinine readings to the age- and sex-appropriate reference value as employed by the full-age spectrum (FAS) method of Pottel [49] or by correcting for height, sex, and an age-dependent k-value in the CKiD-U25 equation from the Schwartz group [50]. Cystatin C is an anti-proteinase produced at a constant rate by all nucleated cells. Cystatin C production is not age-dependent, and reference values are constant above the age of 1 year when indexed GFR has reached adult levels [51]. Differences in extraglomerular handling and their impact on GFR estimation are summarized in Table 2. Although cysteine proteinases and their antagonist cystatin C play a role in tumor spread and invasion, there is no association between cystatin C concentrations and the activity of a malignancy [52]. This was confirmed in a recent paper by Phillips et al. in children with malignancy [53]. While cystatin C is not an acute phase reactant, large epidemiological studies have demonstrated an association between cystatin C levels and high-sensitivity CRP, possibly reflecting low-grade chronic inflammation and atherosclerosis [54]. Still, this does not affect the use of cystatin C as a kidney function parameter in clinical practice.

**Table 2:** Clinically relevant interactions affecting the choice of GFR marker [92–99]

Condition	Cystatin C	Creatinine	Pathophysiology
High doses of glucocorticoids (± 10 mg of prednisone equivalent or more)	Increase	-	Increased cystatin C synthesis
Untreated hypothyroidism	Decrease	-	Decreased cystatin C synthesis
Untreated hyperthyroidism	Increase	-	Increased cystatin C synthesis
Severe obesity	Increase	-	Increased cystatin C synthesis
Neuromuscular disease	-	Decrease	Decreased creatinine production
Severe malnutrition	-	Decrease	Decreased creatinine production
Active malignancy	-	Decrease	Decreased creatinine production
Severe liver disease	-	Decrease	Decreased creatinine production
Creatine supplement	-	Increase	Increased creatinine production
Drugs excreted via organic anion transporter (e.g. trimethoprim)	-	Increase	Interference with tubular creatinine secretion
Urinoma	-	Increase	Recirculation of filtered creatinine, cystatin C does not reach final urine due to proximal tubular degradation

It should also be noted that the calibration of both the creatinine and the cystatin C assays changed with time. In the last two decades, the development and implementation of international standards have solved this issue [47].

Estimation of GFR from serum concentration of creatinine and/or cystatin C requires eGFR equations, which are developed by relating mGFR to simultaneous measurements of creatinine and/or cystatin C and potential covariates in a polynomial logarithmic model such as the CKiD-U25 equation [50]. The composition of the development population (e.g. age, sex, median GFR, co-morbidity, use of medication) influences the applicability of the eGFR equation to specific patient populations [47]. The CKD stage in the development population affects the accuracy of an eGFR equation in populations with a different CKD stage. This is exemplified when comparing the MDRD and the CKD-Epi equations [55]. As the MDRD equation was developed in individuals with lower GFR, it underestimates GFR when GFR is above 60 ml/min/1.73 m^2^, while CKD-Epi, which was calibrated at a higher GFR, does not.

**Fig. 4:**
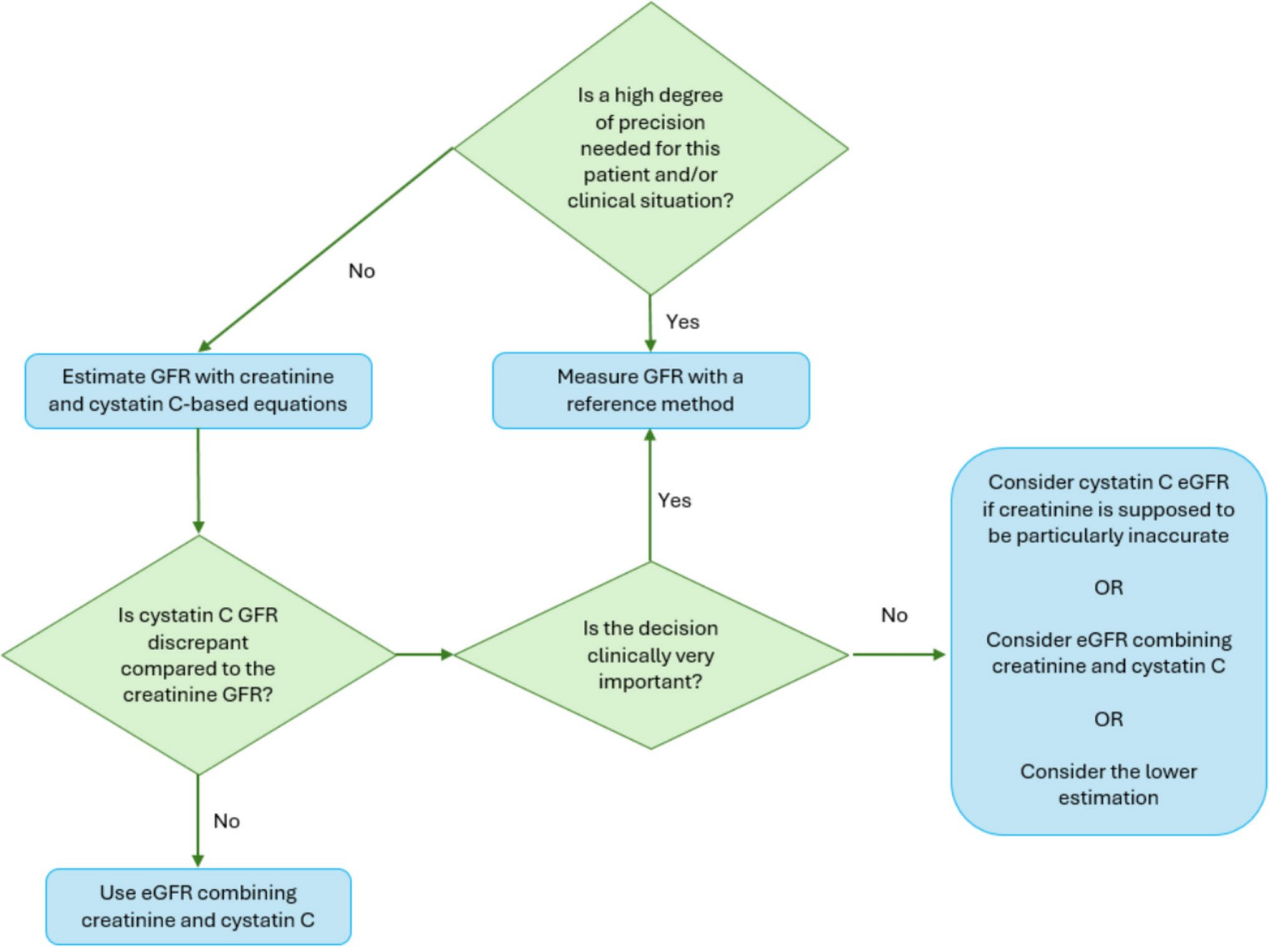
**Measurement of kidney function in clinical practice.** Flowchart giving suggestions on which parameter(s) should be ordered depending on patient characteristics and the clinical situation. Modified from Delanaye et al. [59], used with permission

Besides, there are non-renal factors affecting cystatin C and creatinine metabolism which are not accounted for in eGFR equations (Fig. 3 and Table 2). This explains why the combination equations containing both creatinine and cystatin C [56] or the mean between a cystatin C- and a creatinine-based eGFR perform better than either equation alone [57]. In fact, direct comparison of simultaneous cystatin C- and creatinine-based eGFR gives a good indication of the accuracy of the mean calculated from both results [57]. A large difference exceeding 40% can be an indication to search for extra-renal factors affecting the validity of creatinine or cystatin C in the individual patient [58] and can be an indication to measure GFR directly. This is part of recent recommendations [59] (Fig. 4). Based on the association of an exaggerated elevation of cystatin C compared to creatinine with dismal outcomes in adults, Grubb et al. have proposed the concept of a selective hypofiltration syndrome [60]. In this condition, the rise in cystatin C indicates impaired excretion of macromolecules of comparable size leading to a change in the plasma proteome. There are very few studies on the selective hypofiltration syndrome in children [61, 62]. Also, this condition cannot easily be diagnosed in patients with a malignancy as its definition relies on an *otherwise unexplained* discrepancy between cystatin C and creatinine, a pre-requisite which is not met in many of these patients. Of note, a recent study in adult long-term cancer survivors did not find an increased prevalence in cancer survivors [63].

### Imprecision of eGFR

While eGFR equations perform well on a population level, accuracy on an individual level is rather poor. This is illustrated by the wide scatter between eGFR and mGFR (Fig. 2). Inker translated these data for clinical decision making by visualizing the 90% confidence interval of a given mGFR or eGFR (Fig. 3) [64]. The clinician must realize that an eGFR result of 30 ml/min/1.73 m^2^ means that it is very likely that the patient’s GFR will be between 20 and 40. At normal GFR, the spread is even higher. A P_30_ accuracy above 90% and P_10_ above 50% is regarded as the maximum that can be achieved [47] even when combining both markers [65].

Rowe et al. compared the intraindividual coefficient of variation of iohexol with creatinine and/or cystatin C based CKD-Epi [15]. Of note, the variability of mGFR (6.7%) was higher than the variability of the eGFR equations (5.0 to 5.3%). This probably reflects variation in true GFR and the more complex procedure of mGFR measurement, while the fluctuation of endogenous markers is less marked due to their large volume of distribution acting as a buffer for short-term changes in GFR [48]. Assuming that the extra-renal determinants of cystatin C and creatinine are patient-specific and do not change in the short term, the imprecision of eGFR in the daily follow-up of a given patient is less than the imprecision on a population level. Based on Rowe’s data, a change in eGFR exceeding about 10 to 12% is significant. This is far less than the definition of acute kidney injury [66]. Determining the bias of the eGFR equation in a given patient may be an indication for mGFR measurement, in particular if there is an unexplained discrepancy between creatinine and cystatin C [42, 59]. This helps in judging eGFR values during follow-up.

## GFR estimation in pediatric oncology

Extra-renal factors affecting accuracy in oncological patients in particular are decreased muscle mass or the use of corticosteroids as part of their treatment (Table 2). Indeed, equations established in general populations perform relatively poorly in pediatric oncology patient groups [52, 53, 67–72]. Still, these studies vary in mGFR method, outcome measure, and in the choice of eGFR equations that are compared. This makes it difficult to compare the different equations in this population. In general, eGFR equations using creatinine alone overestimate GFR, resulting in low accuracy, and are outperformed by cystatin C-containing equations [52, 53, 67, 69, 72, 73].

**Table 3:** eGFR equations developed for pediatric oncology patients

Study	Equation	n	mGFR method	mGFR [ml/min/1.73 m^2^]	Age [years]	Diagnoses
Brandt 2003 [74]	eGFR = k × √((age + 6) × wt/Scr) eGFR [ml/min] k = 1.05 for males, 0.95 for females age [months] wt [kg] Scr [mg/dl]	111	Infusion iothalamate	101 (< 1 year) 126 (> 2 years) (mean)	7.95 (mean)	Solid tumors
Cole 2004 [75]	eGFR = 36.76 + 1.91 × wt −0.47 × sCr eGFR [ml/min/1.72 m^2^] wt [kg] sCr [µmol/l]	50	^51^Cr-EDTA Plasma clearance 3 samples	74 (median)	10.5 (median)	Solid tumors
Millisor 2017 [76]	eGFR = 0.748 × (ht/sCr)^0.870^ × Age^0.063^ × (1/BUN)^0.104^ × 1.092^male^ eGFR [ml/min/1.72 m^2^] ht [cm] sCr [mg/dL] age [years] BUN [mg/dL] Male = 1 for yes, 0 for no	1044	^99m^Tc-DTPA plasma clearance 3 samples	114 (median)	6.1 (median)	38% hematological, 40% solid, 18% CNS, 4% multiple
Millisor 2017 simplified [76]	eGFR = 0.33 × (ht/sCr) eGFR [ml/min/1.72 m^2^] ht [cm] sCr [mg/dL]	1044	^99m^Tc-DTPA plasma clearance 3 samples	114 (median)	6.1 (median)	38% hematological, 40% solid, 18% CNS, 4% multiple
Lambert 2021 [77]	eGFR = 77.5 × (sCr/42.25)^−0.423^ × (sCys/0.76)^−0.267^ × (wt/34.3)^0.934^ eGFR [ml/min/1.72 m^2^] sCr [µmol/l] sCys [mg/l] wt [kg]	40	^51^ Cr-EDTA Plasma clearance 4 samples	77.15 (mean)	10.62 (mean)	Solid tumors

In order to try to overcome these challenges, equations have been developed in pediatric oncological populations [74–77] (Table 3), or established eGFR equations have been adapted to fit oncological populations [73]. With the exception of the 5-SJ equation from Millisor et al. [76], group sizes were small and restricted to solid tumors, limiting their generalizability. Also, external validation studies of these equations are scarce [53, 67, 68]. The largest study comes from Phillips et al. [53], who compared two oncological equations (i.e., the creatinine and urea-based 5-SJ equation [76] and the cystatin C-based CysPed equation [67]) with the CKiD Creat-Cys-Urea [56] and the most recent CKiD-Creat-Cys-U25 equation [50]. The authors concluded that all five equations perform comparably. Therefore, equations established in pediatric oncology patients are not significantly better than the general equations. The highest accuracy is seen in equations combining creatinine and cystatin C, reaching P_30_ accuracy rates of 80% and higher [53, 57], in particular in patients with CKD stage 2 and higher. Of note, the Brandt equation performs poorly in several studies [68, 71, 76].

### GFR in children with malignancy

In adult patients, GFR before the start of anti-neoplastic treatment is comparable to age-matched controls, indicating that cancer has no direct impact on kidney function [78]. There are no studies systematically assessing kidney function in children with malignancy per se. Existing data come from studies describing GFR in the context of development or validation of eGFR equations. Ignoring this potential bias, the weighted mean GFR calculated from seven studies describing 1,617 GFR measurements was 112 ml/min/1.73 m^2^ indicating that kidney function is normal in the majority of children undergoing treatment for a malignancy [52, 68, 69, 74–77]. The incidence of significant kidney injury (GFR < 60 ml/min/1.73 m^2^) ranged from 2.9% to 10%. Only Cole et al. reported a significantly higher incidence of 35% [75].

Long-term studies recently reported CKD stage 3 and higher in 2.1 and 3.5% of childhood cancer survivors and kidney failure in 1.1 and 1.7%, respectively. Nephrectomy, abdominal radiation, ifosfamide, and cisplatin were significant treatment-related risk factors [6].

## Drug dosing based on kidney function

Knowledge of kidney function is essential for dosing of drugs which are eliminated primarily by the kidneys. This applies in particular to carboplatin, which has a small therapeutic window between underdosing leading to impaired anti-tumor efficacy and overdosing resulting in toxicity [79, 80]. The linear relationship between carboplatin clearance and GFR initiated the development of carboplatin dosing equations based on desired drug exposure (area under the concentration-time curve; AUC) and the mGFR in adults (Calvert equation) [81] as well as in children (Newell equation) [82]. Renal function-based dosing results in more reproducible and reliable drug exposure than anthropometric dosing [80].

In order to allow for bedside dosing, replacing mGFR with an eGFR in the Newell equation would be most welcome. This has been tested in children by Millisor et al., who compared predicted carboplatin dose derived from mGFR with the results of different eGFR equations [76]. Defining equal drug exposure as less than ± 10% difference in dose determined by mGFR vs. eGFR, the Millisor equation resulted in equal dosing in 40.8% of patients, which was much better than other creatinine-based equations. In a small study in patients with retinoblastoma by van de Velde et al., cystatin C-based equations performed better than weight and creatinine-based equations [83].

Instead of entering eGFR calculated from creatinine and/or cystatin C into the Calvert equation, Thomas et al. developed a dosing equation using both parameters directly [84]. While attractive at first sight, as this method eliminates the additive variability of two successive predictions, Thomas’ model performed worse than the combination of CKD-Epi-Cys-Creat and the Calvert equation [85]. Veal et al. have demonstrated that real-time monitoring with adaptive dosing leads to more consistent platinum exposure in children [86]. This approach determining carboplatin exposure directly might be a valid alternative to performing mGFR if it is possible to measure carboplatin at short notice.

## Conclusion

Fortunately, kidney function is normal in the vast majority of children treated for a malignancy. Following recent guidelines, both in children [87] and in adults [88], monitoring of kidney function should be based on eGFR equations, ideally using both serum creatinine and cystatin C. Clinicians must be aware of the imprecision of eGFR estimates and may need to perform an mGFR measurement, either iohexol or ^99m^Tc-DTPA *plasma clearance* depending on local practice and availability, if the exact knowledge of kidney function is necessary for clinical decision making.

## Supplementary Information

Below is the link to the electronic supplementary material.

ESM 1 Graphical abstract (177 KB PPTX)
