# Supplementary material for: Gold Standard GFR measurement and GFR estimation in pediatric oncology – indications and limitations
Source: Pediatr Nephrol. 2025 Dec 28;41(9):2741–52. doi: 10.1007/s00467-025-07099-0 (PMC13424234; doi:10.1007/s00467-025-07099-0)
Supplement: Supplementary file 1 — Graphical abstract (177 KB PPTX) [file 467_2025_7099_MOESM1_ESM.pptx]

## Slide 1
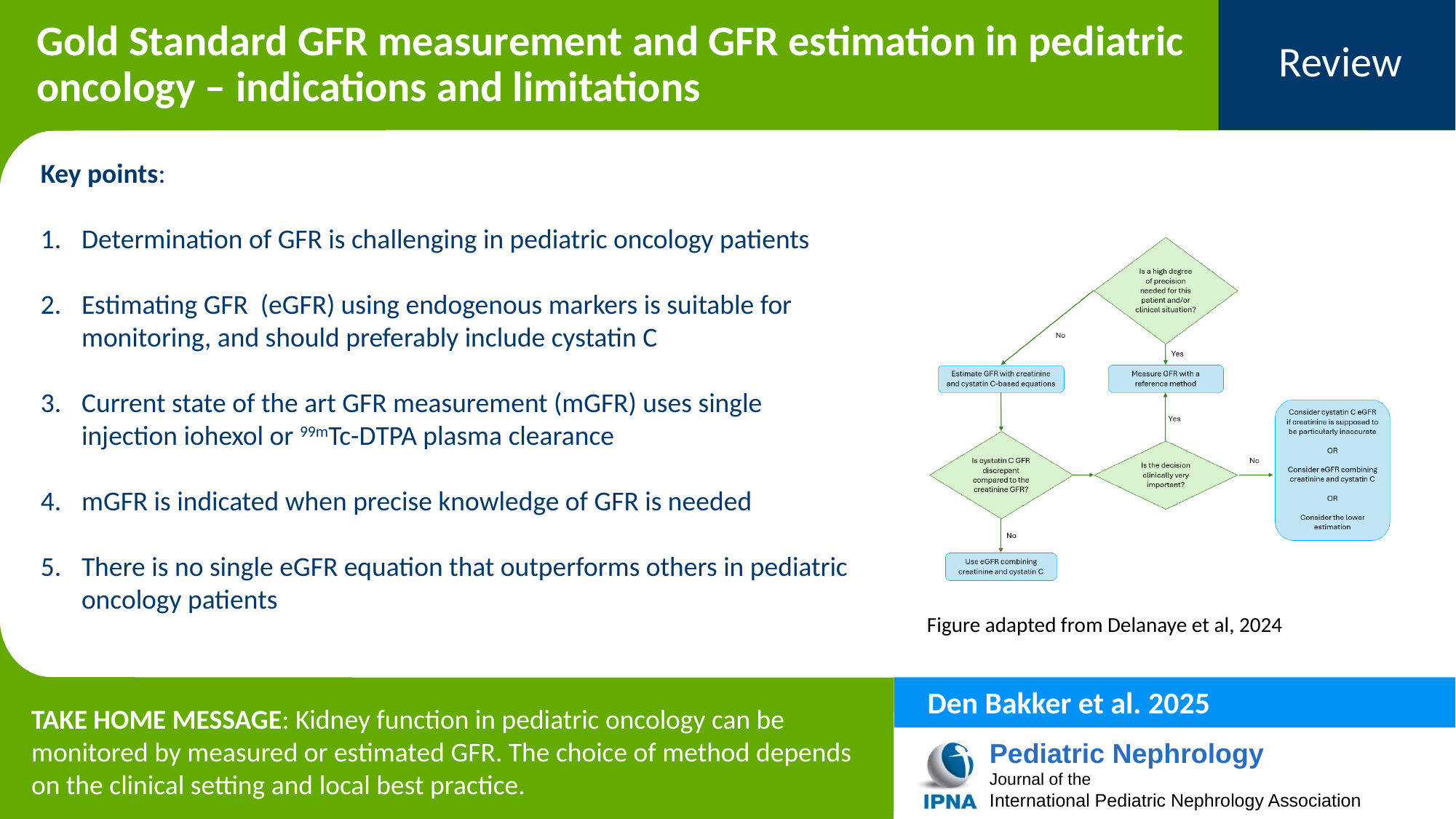

Gold Standard GFR measurement and GFR estimation in pediatric oncology – indications and limitations
Key points:
Determination of GFR is challenging in pediatric oncology patients
Estimating GFR (eGFR) using endogenous markers is suitable for monitoring, and should preferably include cystatin C
Current state of the art GFR measurement (mGFR) uses single injection iohexol or 99mTc-DTPA plasma clearance
mGFR is indicated when precise knowledge of GFR is needed
There is no single eGFR equation that outperforms others in pediatric oncology patients
Figure adapted from Delanaye et al, 2024
Den Bakker et al. 2025
TAKE HOME MESSAGE: Kidney function in pediatric oncology can be monitored by measured or estimated GFR. The choice of method depends on the clinical setting and local best practice.
